# Wastewater Analyses for Psychoactive Substances at Music Festivals: A Systematic Review

**DOI:** 10.1192/j.eurpsy.2026.10470

**Published:** 2026-07-31

**Authors:** R. Cainamisir, X. Zeng, S. B. Himmerich, H. Himmerich

**Affiliations:** Psychological Medicine, King’s College London, London, United Kingdom

## Abstract

**Introduction:**

Music festivals have emerged as venues for the consumption of recreational drugs and novel psychoactive substances (NPS), yet comprehensive surveillance remains challenging.

**Objectives:**

This systematic review provides the first critical evaluation and synthesis of wastewater analysis methods for detecting psychoactive substances at music festivals worldwide.

**Methods:**

Following Preferred Reporting Items for Systematic Reviews and Meta-analyses (PRISMA) guidelines, we systematically searched PubMed, Embase, and MEDLINE databases using terms combining music festivals, drugs, and wastewater analysis.

**Results:**

22 studies (2013-2024) were included, spanning festivals with 6,200 to 600,000 attendees across multiple continents. Two primary sampling approaches emerged: wastewater sampling (15 studies) and pooled urine sampling (7 studies), using LC-MS/MS (Liquid Chromatography–Tandem Mass Spectrometry) and LC-HRMS (Liquid Chromatography–High-Resolution Mass Spectrometry) for chemical analysis. MDMA (3,4-Methylenedioxymethamphetamine), commonly known as ecstasy, emerged as the most consistently detected substance across diverse geographical contexts. Regional variations included a dominance of methamphetamine in Eastern European, a high prevalence of cocaine use in South America, and MDMA use in Western Europe. The music genre significantly influenced consumption patterns, with electronic dance music (EDM) events showing markedly higher MDMA rates.

**Conclusions:**

Limitations include geographic underrepresentation, gender bias in pooled urine sampling, and limited reference standards for NPS. Future research should work on enhanced analytical capabilities and evidence-based harm reduction strategies.

**Disclosure of Interest:**

None Declared

